# Impact of induced magnetic field on Darcy–Forchheimer nanofluid flows comprising carbon nanotubes with homogeneous-heterogeneous reactions

**DOI:** 10.1016/j.heliyon.2024.e24718

**Published:** 2024-01-20

**Authors:** Seemab Bashir, Ibrahim M. Almanjahie, Muhammad Ramzan, Ammara Nawaz Cheema, Muhammad Akhtar, Fatimah Alshahrani

**Affiliations:** a Department of Mathematics, Air University, 44000, Islamabad, Pakistan; b Department of Mathematics, College of Science, King Khalid University, Abha 62223, Saudi Arabia; c Department of Computer Science, Bahria University, Islamabad, 44000, Pakistan; d Fast School of Management, National University of Computer & Emerging Sciences, A.K. Brohi Road, H-11/4, Islamabad, Pakistan; e Department of Mathematical Sciences, College of Science, Princess Nourah bint Abdulrahman University, P.O. Box 84428, Riyadh, 11671, Saudi Arabia

**Keywords:** Heat and mass transfer, Induced magnetic field, Darcy–Forchheimer, MWCNTs and SWCNTs, Homogeneous-heterogeneous chemical reactions

## Abstract

The appealing traits of carbon nanotubes (CNTs) encompassing mechanical and chemical steadiness, exceptional electrical and thermal conductivities, lightweight, and physiochemical reliability make them desired materials in engineering gadgets. Considering such stimulating characteristics of carbon nanotubes, our goal in the current study is to scrutinize the comparative analysis of Darcy–Forchheimer nanofluid flows containing CNTs of both types of multi and single-wall carbon nanotubes (MWCNTs, SWCNTs) immersed into two different base fluids over a stretched surface. The originality of the model being presented is the implementation of the induced magnetic field that triggers the electric conductivity of carbon nanotubes. Moreover, the envisioned model is also analyzed with homogeneous-heterogeneous (h-h) chemical reactions and heat source/sink. The second-order slip constraint is assumed at the boundary of the surface. The transmuted high-nonlinearity ordinary differential equations (ODEs) are attained from the governing set of equations via similarity transformations. The bvp4c scheme is engaged to get the numerical results. The influence of different parameters is depicted via graphs. For both CNTs, the rate of heat flux and the surface drag coefficient are calculated using tables. It is highlighted that an increase in liquid velocity is witnessed for a varied counts volume fraction of nanoparticles. Also, Single-wall water-based carbon nanotube fluid has comparatively stronger effects on concentration than the multi-walled carbon nanotubes in water-based liquid. The analysis also indicates that the rate of heat flux and the surface drag coefficient are augmented for both SWCNTs and MWCNTs for different physical parameters. The said model is also validated by comparing it with a published result.

## 1 Introduction

Nanoscience and nanotechnology are now recognized to be the most stimulating study disciplines, bringing significant advancements in engineering. Abundant nanoparticles and fluid mixtures have extensive uses in sundry industrial developments such as ceramics, food sectors, paints, medication systems, and coatings because of their remarkable properties. Metals such as Silver (Ag), Iron (Fe) , and Aluminum (Al) are used as well as ceramic carbide materials, such as Titanium Carbide (TiC) and Silicon Carbide (SiC). In general, nanofluids are advantageous for enhancing the thermal stability of conventional fluids. CNTs are composed of carbon atoms of cylindrical shapes with a diameter between 0.7 and 50 nm. The SWCNTs comprise a single layer whereas in MWCNTs many layers of graphene are present. These are employed in a variety of applications such as nano-porous filters, radio antennas, electromagnetic gadgets, catalyst supports, electrostatic dissipation, etc. Choi et al. [1] investigated the improvement of thermal conductance in oil-based carbon nanotubes. They found that the nanofluid comprising CNTs exhibits stronger thermal conductance as compared to customary fluids. Nadeem et al. [2] emphasized the significance of MWCNTs for hybrid nanofluid flow in a wavy rectangular duct using the eigenvalue expansion method for improved thermal conductivity. In comparison to the liquid flow (SWCNT/water) model, it is discovered that the pressure gradient accumulates promptly for the hybrid (SWCNT + MWCNT/water) nanofluid model. Additionally, the temperature distribution achieves greater magnitudes for the hybrid (SWCNTs + MWCNTs/water model) phase flow model than for the SWCNT/water model. Zia et al. [3] highlighted the importance of carbon nanotubes considering dissipation and ohmic heating effects using a curved extending sheet. In their work, it is noticed that multi-wall carbon nanotubes (MWCNT) work more efficiently in terms of drag force. Xue model is used to discuss the nanofluid flow with CNTs by Haq et al. [4]. A large local Nusselt number is observed for higher engine oil-based CNT in this case. Both single and multi-wall characteristics of CNTs are considered in a mixed convective flow by Hayat et al. [5]. Findings revealed that a rise in the number of carbon nanotubes lowers heat transmission. Khan et al. [6] explained the physical aspects of SWCNTs and MWCNTs flow combined with induced flux and quartic chemical reactions. Results exhibit that the surface drag coefficient shows a lowered trend against larger counts of velocity ratio parameter. Also, velocity is boosted through nanoparticle volume fraction. Several researchers [[7], [8], [9], [10], [11], [12], [13]] explained other diverse features of nanofluids and CNTs.

Magnetohydrodynamics (MHD) delves into the investigation of the induction of a magnetic field in a fluid flow. It happens when a magnetic field flows through a liquid metal or other electrically conducting fluid, such as plasma. An induced magnetic field is created within the liquid as a consequence of the interaction between the velocity of the fluid and magnetic flux. The fluid's internal magnetic field also the outside of the liquid may interact in the induced magnetic field. The resulting magnetic field is a result of the interaction between the fluid's electric currents and the external magnetic field. The behavior of the conducting liquid can be significantly affected by the induced magnetic field. Induced magnetic field possesses numerous applications including electromagnetic propulsion, medical imaging, security and mining, material testing, and navigation. Many scientists [[14], [15], [16], [17]] in the disciplines of medical sciences and research have been interested in MHD. Upreti et al. [18] inspected the induced magnetic field and assessed the thermal transmission for the blood-based nanoliquid while considering the modified Fourier model. The academics observed that when the velocity slip parameter improves, so do the induced magnetic field distributions. Iqbal et al. [19] used an induced magnetic field to elaborate the transport process in the presence of CNTs and bioconvection nanoparticles. They emphasized that when the magnetic field is enhanced MWCNTs contribute more than SWCNTs. With the role of thermal radiative flux, Joshi et al. [20] described the magnetized flow of Casson hybrid nanoliquid due to a Riga surface. The observations demonstrate that the Xue model improved thermal conductivity distribution is superior to that of the Hamilton-Crosser model. Hayat et al. [21] discussed the magnetic field in a radial direction in the presence of Carreau-Yasuda fluid movement. They perceived that the velocity and temperature upsurged with increasing MHD.

Many authors have focused a great deal of emphasis on chemical processes in fluid mechanics [[22], [23], [24], [25], [26], [27]]. The relevance of chemical reactions in the movement of liquids is demonstrated by a wide range of applications, such as the hydrometallurgical industries, polymer manufacture, food processing, ceramics, fog creation, fog dispersion, and freezing effects in crop damage. There are two categories of chemical reactions: homogeneous and heterogeneous. Every reactant in a homogeneous reaction is in the same phase or state of matter. This indicates that either all of the reactants are in the liquid, solid, or gas phases. The reactants in a heterogeneous reaction are in many phases or states of matter. This indicates that one or more of the reactants are not in the same state as the others—that is, solid, liquid, or gas. The terms “homogeneous” and “heterogeneous” are used in fluid mechanics to characterize the characteristics and makeup of a fluid or fluid system. In order to optimize the process and get important insights into the behavior of reactions in fluid settings, both h-h reactions are included concurrently in many chemical processes. Ramzan et al. [28] discussed the consequence of modified Fourier law effects on Cross-Hybrid Nanofluid with Autocatalytic Chemical Reaction. They noticed the concentration of the hybrid nanoliquid becomes lower due to the enhancement of values of chemical reaction, thermophoresis, and Schmidt number. Bashir et al. [29] address the comparison of five nanoparticles in the presence of h-h type processes. When the estimates of h-h reactions rise, the concentration boundary layer for all five nanoparticles is observed to diminish.

Given the foregoing, it is witnessed that enormous studies can be mentioned that have discussed the impact of MHD with h-h reactions, and in all these researches, the impression of the induced magnetic flux (IMF) is omitted. However, the impact of induced magnetic flux is being considered in the present investigation. The other factors added to the manuscript are second-order slip and Darcy-Forchheimer effects. Also, homogeneous-heterogeneous reactions are considered with the second-order velocity slip condition which is incorporated into the boundary. The comparative analysis is made of SWCNTs and MWCNTs immersed in water (H_2_O) and Kerosene oil as the base liquids. The impact of different arising parameters on the flow, temperature, and concentration is calculated. The phenomena of transport are described by governing equations that take into account the effects of nonlinear heat sources and sinks. Existing complex nonlinear equations are numerically handled by MATLAB bvp4c. The various parameters of behavior are deliberated via graphical findings. To get confidence in the reliability, and uniqueness of the presented model, a comparison with published works is presented in Table 1. Supplementary the current model's main goal is to provide answers to the following queries:

➢ How is the behavior of the momentum boundary layer exposed in terms of the volume fraction of the nanoparticle?
➢ What are the consequences of first and second-order slip boundary constraints on the fluid velocity?
➢ What are the major effects on the fluid temperature by introducing heat source and sink conditions?
➢ What is the association between the induced magnetic flux and reciprocal magnetic Prandtl number?
➢ How is the concentration boundary layer affected by the homogeneous reaction parameter?
➢ Which nanofluid with different base fluid combinations is more influential?
➢ What impact of the volume fraction is noticed on the Skin friction and heat transmission rate?

**Table 1:** A comparison of present work with closely comparable published research efforts.

References	Darcy–Forchheimer	Homogeneous/Heterogeneous Reaction	2nd order velocity slip condition	Tiwari Das model	InducedMagnetic Flux	Heat source/Sink	SWCNTs/MWCNTs
[4]	Yes	No	No	No	No	No	Yes
[6]	No	Yes	No	Yes	Yes	No	Yes
[27]	No	No	No	No	Yes	Yes	Yes
[19]	No	Yes	No	Yes	No	Yes	Yes
Present	**Yes**	**Yes**	**Yes**	**Yes**	**Yes**	**Yes**	**Yes**

## 2 Mathematical modeling

The following are the assumptions for the envisioned mathematical model:

➢ An incompressible steady flow comprising CNTs towards the stretching sheet is considered.
➢ Suspension of SWCNTs and MWCNTs is assumed in H_2_O and Kerosene oil-based fluids.
➢ The current investigation is based on the assumption of homogeneous and heterogeneous reactions together with a heat sink and source term for the Darcy-Forchheimer flow.
➢ The surface is stretched on the x-axis and the area y>0 is inhabited by nano liquid. Let's postulate $u_{e}=ax$ and $u_{w}=cx$.
➢ Induced magnetic field H is applied. Where $H_{1}$ and $H_{2}$ are parallel and perpendicular components of H.
➢ second-order velocity slip condition is incorporated into the boundary.

**Fig. 1:**
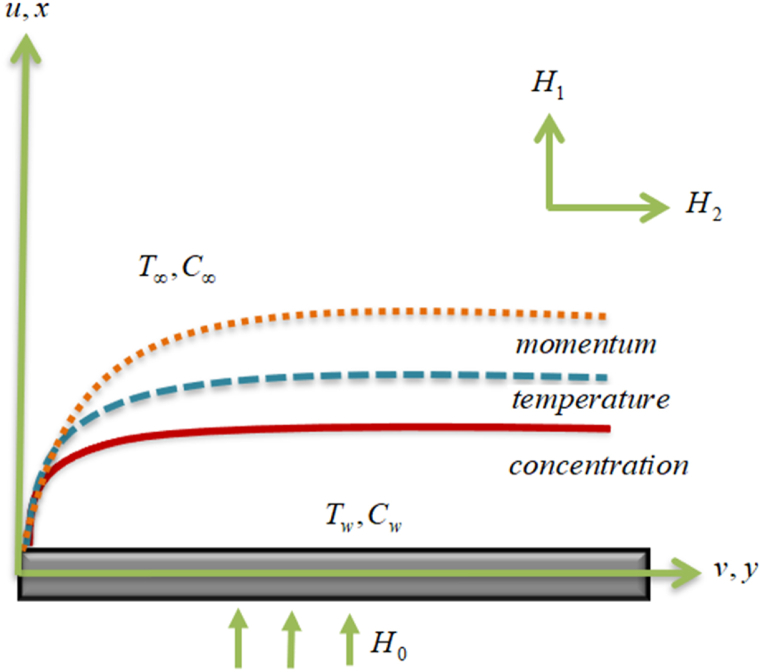
Sketch of the flow model.

The outline of the flow is illustrated in Fig. 1.

The subsequent chemical reaction [22] is considered:

(1) $$A_{1}+2B_{1}\to 3B_{1},Rate=k_{1}C_{1}{C_{2}}^{2}\text{.}$$

Let us take an isothermal chemical reaction:

(2) $$A_{1}\to 3B_{1},Rate=k_{2}C_{1}\text{.}$$

Model equations are written as follows under the aforementioned assumptions [[4], [5], [6],^22^]:

(3) $${\overset{⌢}{u}}_{x}+{\overset{⌢}{v}}_{y}=0\text{,}$$

(4) $${\left({\overset{⌢}{H}}_{1}\right)}_{x}+{\left({\overset{⌢}{H}}_{2}\right)}_{y}=0\text{,}$$

(5) $$\overset{⌢}{u}{\overset{⌢}{u}}_{x}+\overset{⌢}{v}{\overset{⌢}{u}}_{y}-\frac{\mu_{e}}{4\pi \rho_{nf}}\left[{\overset{⌢}{H}}_{1}{\left({\overset{⌢}{H}}_{1}\right)}_{x}+{\overset{⌢}{H}}_{2}{\left({\overset{⌢}{H}}_{1}\right)}_{y}\right]=\frac{\mu_{e}}{4\pi \rho_{nf}}{\overset{⌢}{H}}_{e}{{\overset{⌢}{H}}_{1}}^{\prime }+\frac{\mu_{nf}}{\rho_{nf}}{\overset{⌢}{u}}_{yy}-\frac{\mu_{nf}}{\rho_{nf}k^{*}}\overset{⌢}{u}-F{\overset{⌢}{u}}^{2}\text{,}$$

(6) $$\overset{⌢}{u}{\left({\overset{⌢}{H}}_{1}\right)}_{x}-{\overset{⌢}{H}}_{1}{\overset{⌢}{u}}_{x}+\overset{⌢}{v}{\left({\overset{⌢}{H}}_{2}\right)}_{y}-{\overset{⌢}{H}}_{2}{\overset{⌢}{u}}_{y}=\mu_{e}{\left({\overset{⌢}{H}}_{1}\right)}_{yy}\text{,}$$

(7) $$\overset{⌢}{u}{\overset{⌢}{T}}_{x}+\overset{⌢}{v}{\overset{⌢}{T}}_{y}=\alpha_{nf}{\overset{⌢}{T}}_{yy}+\frac{q*}{{\left(\rho c_{p}\right)}_{nf}}\text{,}$$

(8) $$\overset{⌢}{u}{\left(C_{1}\right)}_{x}+\overset{⌢}{v}{\left(C_{1}\right)}_{y}=D_{A}{\left(C_{1}\right)}_{yy}-K_{1}C_{1}{C_{2}}^{2}\text{,}$$

(9) $$\overset{⌢}{u}{\left(C_{2}\right)}_{x}+\overset{⌢}{v}{\left(C_{2}\right)}_{y}=D_{B}{\left(C_{2}\right)}_{yy}+K_{1}C_{1}{C_{2}}^{2}\text{,}$$

A dimensional analysis of the above governing model equations is presented in Appendix A section, at the end of the paper.

Particularly at small scales or with non-traditional surface qualities, the second-order slip condition is thought to account for slip effects at the fluid-solid interface and can significantly affect fluid profiles in certain scenarios. When a more precise depiction of fluid behavior close to the solid boundary is needed, a second order slip boundary condition which is more sophisticated than the no-slip condition is employed.

Considering the following set of boundary conditions [[4], [5], [6],^27^]:

$$\begin{array}{l} \overset{⌢}{u}=u_{slip}+cx,{\left({\overset{⌢}{H}}_{1}\right)}_{y}=0,\overset{⌢}{v}=0,{\overset{⌢}{H}}_{2}=0,D_{A}{\left(C_{1}\right)}_{y}=K_{2}C_{1}\text{,}\\ D_{B}{\left(C_{2}\right)}_{y}=-K_{2}C_{1},\overset{⌢}{T}=T_{w}\;at\;y=0\text{,} \end{array}$$

(10) $$\overset{⌢}{u}\to ax=u_{e},\overset{⌢}{T}\to T_{\infty },{\overset{⌢}{H}}_{1}\to H_{o}x=H_{e}\left(x\right),C_{1}\to C_{o},C_{2}\to 0\;as\;y\to \infty$$

where slip velocity $u_{slip}$ is given by Ref. [27]:

(11) $$u_{slip}=\frac{2}{3}\left(\frac{3-\xi m^{3}}{\xi }-\frac{3}{2}\frac{1-m^{2}}{k_{n}}\right)\omega \;{\overset{⌢}{u}}_{y}=\frac{1}{4}\left[m^{4}+\frac{2}{{k_{n}}^{2}}\left(1-m^{2}\right)\right]\omega^{2}{\overset{⌢}{u}}_{yy}={\overset{⌢}{u}}_{y}{A_{1}}^{*}+{\overset{⌢}{u}}_{yy}{B_{1}}^{*}\text{,}$$

where $m=\min\left[\frac{1}{k_{n}},1\right]\text{,}$ the momentum accommodation coefficient ξ is varying between 0 and 1, $k_{n}$ is the Knudsen number, and ω is the molecular mean free path respectively. $\overset{⌢}{v}=0$ in the boundary condition indicates sheet is not permeable, change in the magnetic flux is zero at the surface. The heterogeneous reaction is deployed between the fluid and the surface of the sheet *i.e*., the wall itself is a catalyst. Far from the surface, free stream velocity $u_{e}$, ambient temperature $T_{\infty }\text{,}$ and ambient concentration $C_{\infty }$ is considered. $F=\frac{c_{b}}{x\sqrt{k^{*}}}$ is used for the non-uniform inertia coefficient of the spongy medium. In the energy equation $q*=\left[A^{*}\left(T_{w}-T_{\infty }\right)f^{\prime }+\left(B^{*}\left(T-T_{\infty }\right)\right)\right]\frac{k_{f}u_{w}}{x\upsilon_{f}}\text{,}$ illustrates the variable heat source and sink with the heat absorption ($A^{*}<0$ and $B^{*}<0$) and the generation of heat ($A^{*}>0$ and $B^{*}>0$). We use the following thermal physical properties for nanofluid with thermal conductivity taken as Xue model [[6], [20]], which is specifically modeled for CNTs:

$$\alpha_{nf}=\frac{k_{nf}}{{\left(\rho c_{p}\right)}_{nf}},{\left(\rho c_{p}\right)}_{nf}=\left(1-\varphi \right){\left(\rho c_{p}\right)}_{f}+\varphi {\left(\rho c_{p}\right)}_{CNT},\mu_{nf}={\left(1-\varphi \right)}^{-2.5}\mu_{f}\text{,}$$

(12) $$k_{nf}=\frac{\left(1-\varphi \right)+2\varphi \frac{k_{CNT}}{k_{CNT}-k_{f}}\ln\frac{k_{CNT}+k_{f}}{2k_{f}}}{\left(1-\varphi \right)+2\varphi \frac{k_{f}}{k_{CNT}-k_{f}}\ln\frac{k_{CNT}+k_{f}}{2k_{f}}}k_{f},\rho_{nf}=\left(1-\varphi \right)\rho_{f}+\varphi \rho_{CNT}\text{.}$$

$$\overset{⌢}{u}=cxf^{\prime }\left(\eta \right),\eta =y\sqrt{\frac{c}{\upsilon_{f}}},{\overset{⌢}{H}}_{1}=H_{o}xg^{\prime }\left(\eta \right),\overset{⌢}{v}=-\sqrt{c\upsilon_{f}}f\left(\eta \right)\text{,}$$

(13) $${\overset{⌢}{H}}_{2}=-\;H_{o}\;\sqrt{\frac{c}{\upsilon_{f}}}g\left(\eta \right),\theta \left(\eta \right)=\frac{\overset{⌢}{T}-T_{\infty }}{T_{w}-T_{\infty }},h\left(\eta \right)=\frac{C_{1}}{C_{ο}},l\left(\eta \right)=\frac{C_{2}}{C_{ο}}\text{.}$$

Eqs. (3), (4) are continuity equations satisfied trivially while the rest of the flow equations are:

(14) $$f^{\prime \prime \prime }-\left(\left(1-\varphi \right)+\varphi \frac{\rho_{CNT}}{\rho_{f}}\right){\left(1-\varphi \right)}^{2.5}\left(\left(1+F_{r}\right){f^{\prime }}^{2}-f^{\prime \prime }\;f\right)-\lambda_{2}f^{\prime }+\beta \left(-1+{g^{\prime }}^{2}-gg^{\prime \prime }\right)=0\text{,}$$

(15) $$\lambda g^{\prime \prime \prime }+g^{\prime \prime }-g\;f^{\prime \prime }=0\text{,}$$

(16) $$\frac{1}{\mathrm{Pr}}\frac{K_{nf}}{K_{f}}\theta^{\prime \prime }+\left(\left(1-\varphi \right)+\varphi \frac{{\left(\rho c_{p}\right)}_{CNT}}{{\left(\rho c_{p}\right)}_{f}}\right)f\theta^{\prime }+\frac{1}{\mathrm{Pr}}\left(A^{*}f^{\prime }+B^{*}\theta \right)\text{,}$$

(17) $$h^{\prime \prime }+Sc\;fh^{\prime }-K\;Sc\;hl^{2}=0\text{,}$$

(18) $$l^{\prime \prime }+\frac{Sc}{\delta^{*}}\;fl^{\prime }-\frac{K\;Sc}{\delta^{*}}\;hl^{2}=0\text{,}$$

with

(19) $$\begin{array}{l} 1+\gamma f^{\prime \prime }\left(0\right)+\delta f^{\prime \prime \prime }=f^{\prime }\left(0\right),f\left(0\right)=0,f^{\prime }\left(0\right)=1,g^{\prime \prime }\left(0\right)=g\left(0\right)=0,\theta \left(0\right)=1\text{,}\\ h^{\prime }\left(0\right)=K^{*}h\left(0\right),\delta^{*}l^{\prime }\left(0\right)=-K^{*}h\left(0\right),f^{\prime }\left(\infty \right)\to A,g^{\prime }\left(\infty \right)\to 1,\theta \left(\infty \right)\to 0\text{,}\\ h\left(\infty \right)\to 1,l\left(\infty \right)\to 0\text{.} \end{array}$$

when $\delta^{*}=1\text{,}$ or $D_{A}=D_{B}=1\text{,}$ we have

(20) l(η)+h(η)=1,

As a result, the previous Equations (17), (18) are replaced by (20) in the following way:

(21) $$h^{\prime \prime }\left(\eta \right)+Sc\;fh^{\prime }-K\;Sc\;h{\left(1-h\right)}^{2}=0\text{,}$$

with boundary condition

(22) $$h^{\prime }=hK^{*},h\left(\infty \right)\to 1\text{.}$$

The dimensionless variables in the equations above are:

(23) $$\begin{array}{l} A=\frac{a}{c},\beta =\frac{\mu_{f}}{4\pi \rho_{f}}\left(\frac{H_{o}}{\upsilon_{f}}\right),\lambda =\frac{\mu_{e}}{\upsilon_{f}},\lambda_{2}=\frac{\upsilon_{f}}{K^{*}c},F_{r}=\frac{C_{b}}{\sqrt{k^{*}}},K=\frac{K_{1}{C_{{}^{\circ}}}^{2}}{c}\text{,}\\ \theta_{w}=\frac{T_{w}}{T_{\infty }},\gamma =A_{1}^{*}\sqrt{\frac{c}{\upsilon_{f}}},\delta =B_{1}^{*}\frac{c}{\upsilon_{f}},\mathrm{Pr}=\frac{{\left(\mu c_{p}\right)}_{f}}{k_{f}},\text{Sc}=\frac{\upsilon_{f}}{D_{A}},\delta^{*}=\frac{D_{B}}{D_{A}}\text{,}\\ K^{*}=\frac{K_{2}\sqrt{\upsilon_{f}}}{D_{A}\sqrt{c}}\text{,} \end{array}$$

where $F_{r}$ is used for the Forchheimer parameter, A is the stretching parameter, β is the magnetic parameter, λ is reciprocal magnetic Prandtl number, $\lambda_{2}$ is the porosity parameter, K is homogeneous reaction parameter, $\theta_{w}$ is temperature ratio parameter, Pr is the Prandtl number, Sc is Schmidt number, $K^{*}$ is the heterogeneous reaction parameter, $\delta^{*}$ is the rate of diffusion coefficient. $\gamma =A_{1}^{*}\sqrt{\frac{c}{\upsilon_{f}}}>0$ is the first-order velocity parameter and $\delta =B_{1}^{*}\frac{c}{\upsilon_{f}}<0$ second-order slip parameter.

The traits of CNTs, water, and Kerosene oil are revealed in Table 2.

**Table 2:** Physical and thermal traits of CNTs [3,4,6].

Physical Features	k(W/mK)	$\rho \left({\text{kgm}}^{-3}\right)$	$c_{p}\left(J/kgK\right)$	Pr
H_2_O	0.613	997.1	4179	6.2
Kerosene oil	0.145	783	2090	23
SWCNTs	6600	2600	425	
MWCNTs	3000	1600	796	

## 3 Quantities of physical interest

### 3.1 Skin friction coefficient

Mathematically

(24) $$C_{f}=\frac{2\tau_{w}}{\rho_{f}{u_{w}}^{2}}\text{,}$$

where

(25) $$\tau_{w}=\mu_{nf}{\left(\frac{\partial u}{\partial y}\right)|}_{y=0}\text{.}$$

(26) $$C_{f}{\text{Re}}_{x}^{1/2}=\frac{1}{{\left(1-\varphi \right)}^{2.5}}f^{\prime \prime }\left(0\right)\text{.}$$

### 3.2 Local Nusselt number

Mathematically, we have

(27) $$Nu=\frac{xq_{w}}{k_{f}\left(T_{w}-T_{\infty }\right)}\text{,}$$

where wall heat flux is

(28) $$q_{w}=-K_{nf}{T_{y}|}_{y=0}\text{,}$$

Utilizing the above equations produces the following result:

(29) $$Nu_{x}{\text{Re}}_{x}^{1/2}=-\left(\frac{K_{nf}}{K_{f}}\right)\theta^{\prime }\left(0\right)\text{.}$$

where ${\text{Re}}_{x}^{1/2}=\frac{cx^{2}}{\upsilon_{f}}$ is the local Reynold number.

## 4 Numerical solution outline

**Fig. 2:**
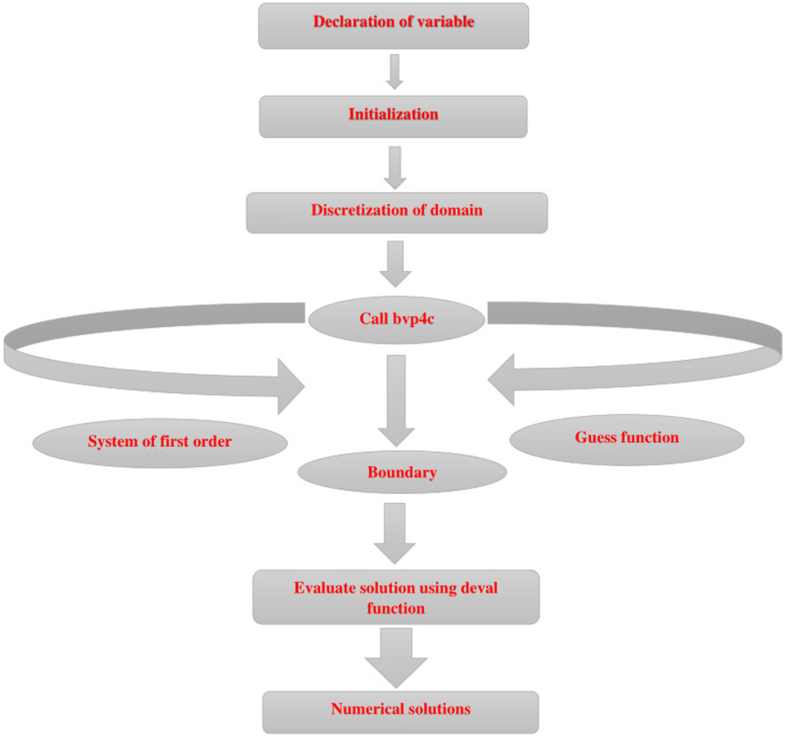
Flow chart of numerical scheme.

The visual representation detailing the numerical method is elucidated in Fig. 2. To evaluate the system of Eqs. (14)–(16) with (19), (21) with (22), MATLAB software bvp4c is employed.

✓ A simpler and faster-to-converge procedure.
✓ Analytically superior to other analytical methods in terms of accuracy.

We generate residuals and perform computations for distinct step sizes h=0.01,0.001,.... via bvp4c. The convergence criteria are taken as $10^{-6}$. It is important that the essential finite values of $\eta_{\infty }$ are picked. The constraints at $\eta_{\infty }$ for the particular case are limited to η=2.5, that is adequate to demonstrate the behavior of the asymptotic solution.

For this reason, new variables are taken for provided as:

$$N=\left(\left(1-\varphi \right)+\varphi \frac{{\left(\rho c_{p}\right)}_{CNT}}{{\left(\rho c_{p}\right)}_{f}}\right),D=\frac{K_{nf}}{K_{f}},H=\left(\left(1-\varphi \right)+\varphi \frac{{\left(\rho c_{p}\right)}_{CNT}}{{\left(\rho c_{p}\right)}_{f}}\right){\left(1-\varphi \right)}^{-2.5}\text{,}$$

(30) $$\begin{array}{l} f\left(\eta \right)=y_{1},g\left(\eta \right)=y_{4},\theta \left(\eta \right)=y_{7}\text{,}\\ f^{\prime }\left(\eta \right)=y_{2},g^{\prime }\left(\eta \right)=y_{5},f^{\prime \prime }\left(\eta \right)=y_{3}\text{,}\\ g^{\prime \prime }\left(\eta \right)=y_{6},f^{\prime \prime \prime }\left(\eta \right)=yy_{1},g^{\prime \prime \prime }\left(\eta \right)=yy_{1}\text{,} \end{array}$$

$$\begin{array}{l} \theta^{\prime }\left(\eta \right)=y_{8},\theta^{\prime \prime }\left(\eta \right)=yy_{3},h\left(\eta \right)=y_{9}\text{,}\\ h^{\prime }\left(\eta \right)=y_{10},h^{\prime \prime }\left(\eta \right)=yy_{4}\text{.} \end{array}$$

The use of the above expressions leads to the subsequent set of first-order problem:

(31) $$yy_{1}=\lambda_{1}y_{2}-\beta \left({y_{5}}^{2}-y_{4}y_{6}-1\right)+H\left(\left(1+F_{r}\right){y_{2}}^{2}-y_{1}y_{3}\right)\text{,}$$

(32) $$yy_{2}=\frac{1}{\lambda }\left(-y_{1}y_{6}+y_{3}y_{4}\right)\text{,}$$

(33) $$yy_{3}=\frac{1}{D}\left(-\mathrm{Pr}\;Hy_{1}y_{8}-\left(A^{*}y_{1}+B^{*}y_{7}\right)\right)\text{,}$$

(34) $$yy_{4}=Sc\left(-y_{1}y_{10}+Ky_{9}{\left(1-y_{9}\right)}^{2}\right)\text{.}$$

with the transformed BCs

(35) $$\begin{array}{l} y_{2}\left(0\right)-\gamma y_{3}\left(0\right)-\delta \left(\left(H\left(1+F_{r}\right)-\lambda_{1}\right)+\lambda_{2}\right)=1,y_{4}\left(0\right)=0,y_{2}\left(0\right)=1,y_{6}\left(0\right)=1\text{,}\\ y_{10}\left(0\right)=K_{1}y_{9}\left(0\right),y_{7}\left(0\right)=1,y_{2}\left(\infty \right)=A,y_{5}\left(\infty \right)=1,y_{7}\left(\infty \right)=0,y_{9}\left(\infty \right)=1\text{.} \end{array}$$

## 5 Graphical discussion

This section highlights the graphical discussion considering different emerging parameters $F_{r},\delta ,\lambda_{2},A^{*},B^{*},K,K_{1}\text{,}$ [4,6,18]. SWCNTs and MWCNTs are physically explained with the help of graphs in the base fluid water and Kerosene oil.

### 5.1 Velocity distribution

**Fig. 3:**
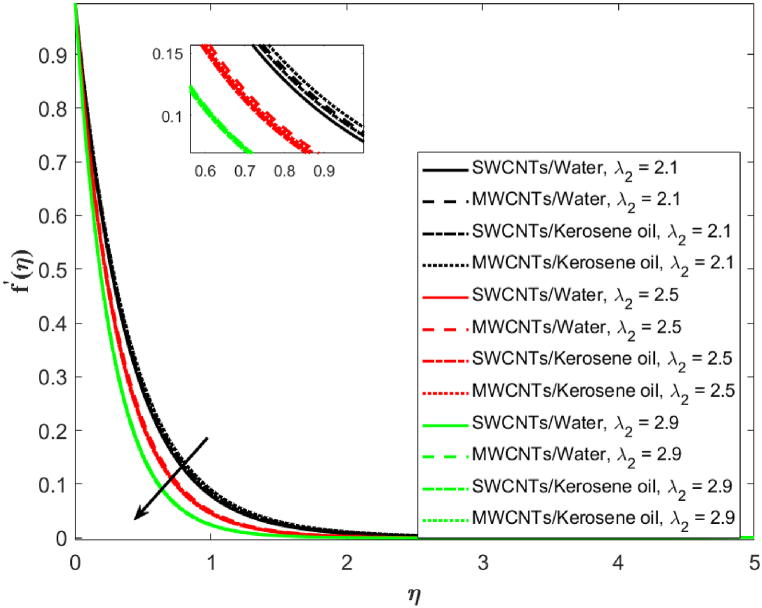
Fluid velocity against porosity parameter $\lambda_{2}$.

**Fig. 4:**
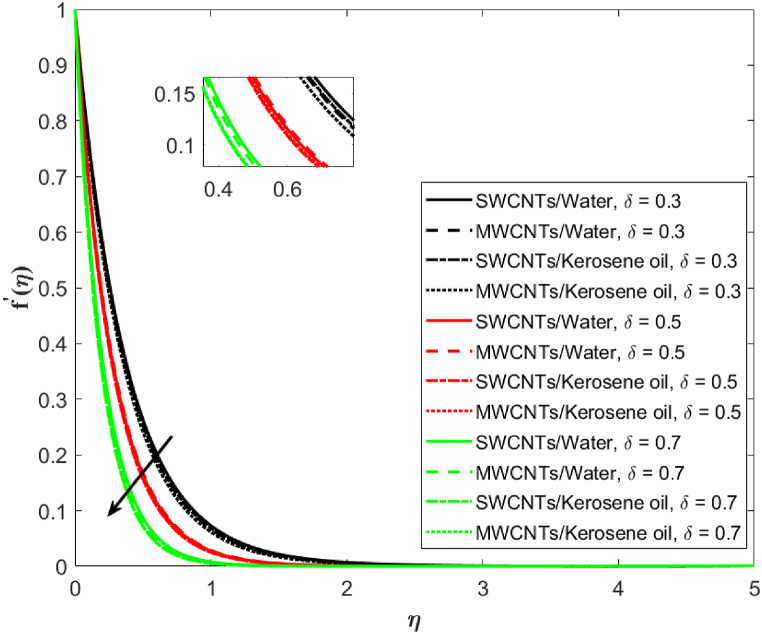
Fluid velocity against second-order velocity slip parameter δ.

**Fig. 5:**
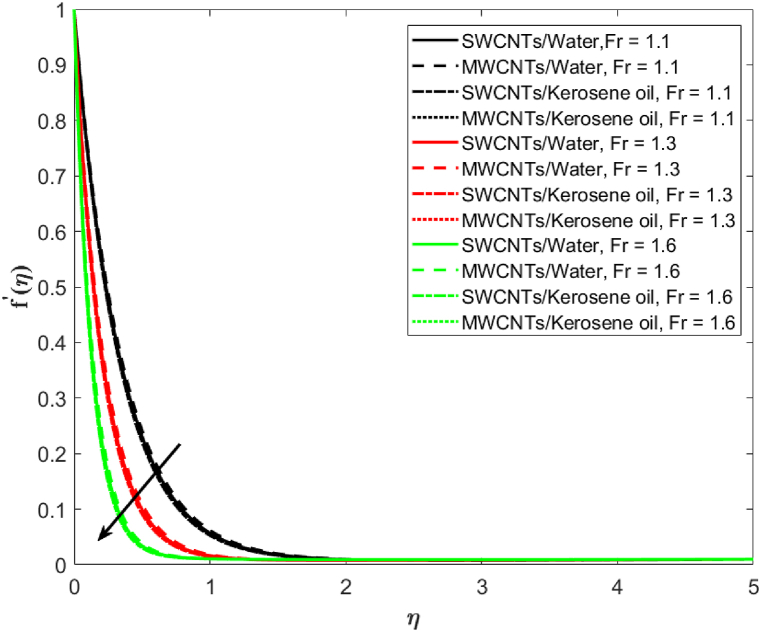
Fluid velocity against Darcy-Forchheimer parameter $F_{r}$.

In Fig. 3, the velocity distribution for MWCNTs and SWCNTs is examined for different values of porosity parameter $\left(\;\lambda_{2}=2.1,2.5,2.9\right)$. Decreasing trends in the velocity are observed on increasing $\lambda_{2}$ for both MWCNTs as well as SWCNTs. MWCNTs respond more strongly than SWCNTs. The hurdle is observed in the liquid flow because of the presence of penetrable space which is responsible for the decrease in velocity. The association of the second-order velocity slip parameter δ and the fluid velocity is depicted in Fig. 4. The mounting behavior of the velocity is captured here for increasing (δ=0.3,0.5,0.7). High resistance is offered to the flow when there is no slip. As the velocity slip parameter improves, fluid resistance decreases which increases the velocity of SWCNTs and MWCNTs. Fig. 5 is drawn to give an idea about the consequence of the Darcy-Forchheimer parameter Fr on the fluid velocity. It is apparent that the growing Forchheimer parameter (Fr=1.1,1.3,1.6) decreases the velocity.

### 5.2 Induced magnetic field and temperature distributions

**Fig. 6:**
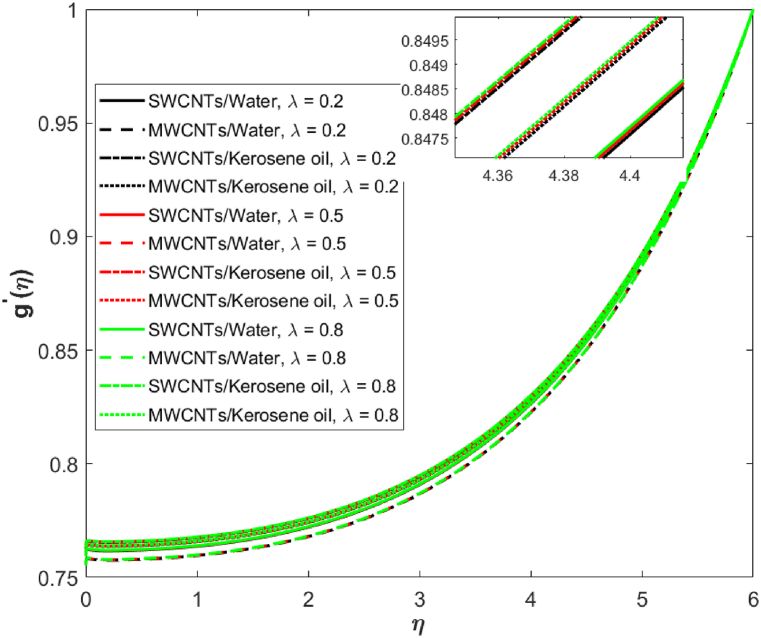
Induced magnetic field against reciprocal Prandtl number λ.

**Fig. 7:**
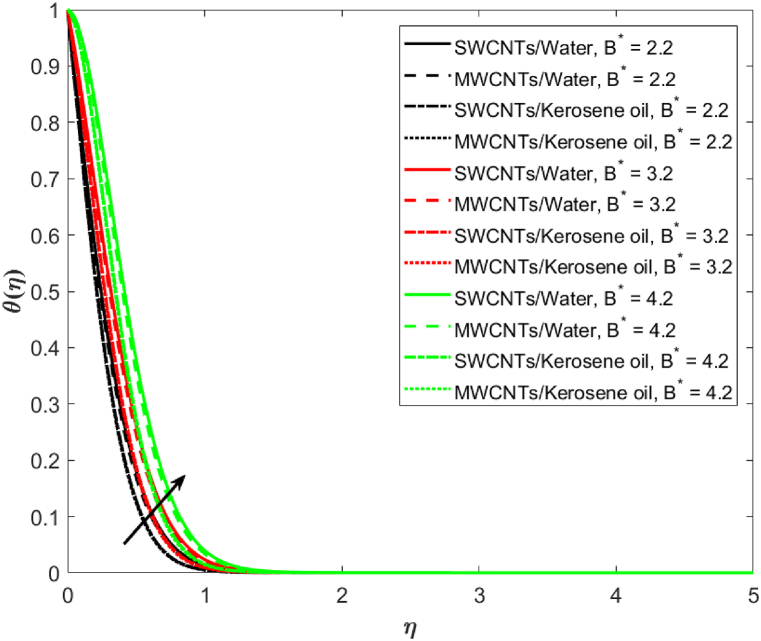
Fluid temperature against $B^{*}$.

The effects of reciprocal Prandtl number λ is highlighted on the Induced magnetic field $g^{\prime }\left(\eta \right)$. Here, $g^{\prime }\left(\eta \right)$ is observed as a decreasing function of λ in Fig. 6. Physically, the larger value of (λ=0.2,0.5,0.8) leads to a higher electric force, and the Lorentz force increases against higher λ , and therefore the induced magnetic field is decreased in both water and kerosene oil-based single-wall and multi-wall CNTs. The effects of heat source/sink coefficient $B^{*}$ versus fluid temperature is expressed in Fig. 7. Basically, the source term **(**$A^{*}>0$ and $B^{*}>0$**)** rises θ(η) because of higher energy. Also, water-based single-wall and multi-wall CNTs have a larger impact on temperature as compared to kerosene oil-based fluid.

### 5.3 Concentration distribution

**Fig. 8:**
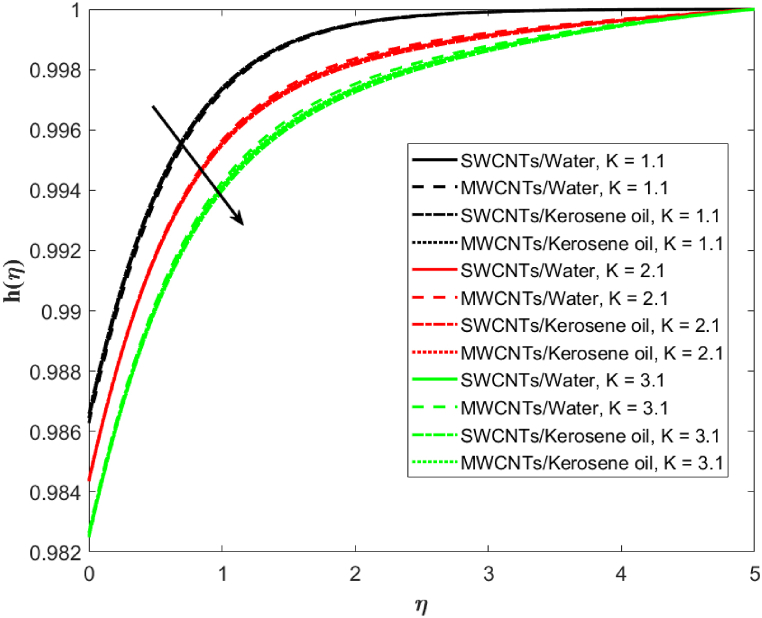
Fluid concentration against homogeneous reaction parameter *K*.

**Fig. 9:**
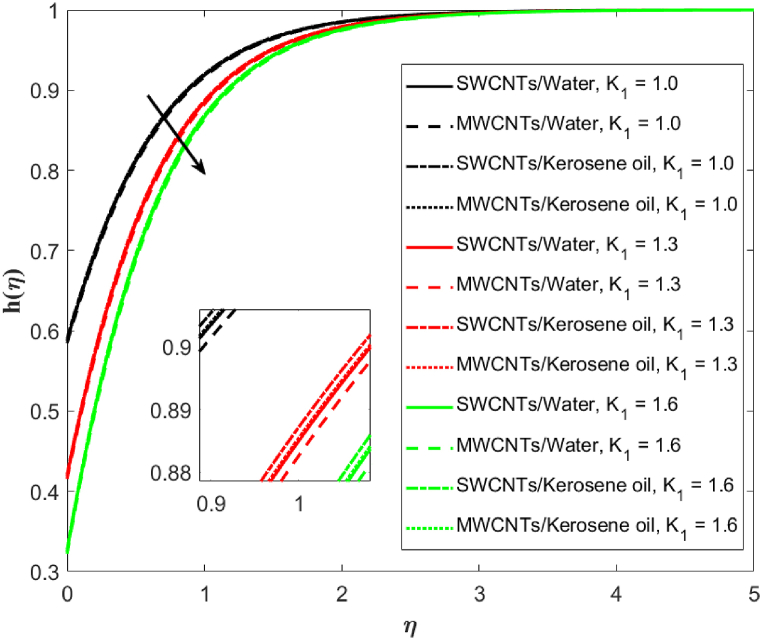
Fluid concentration against rate constant *K*_1_.

The impression of the homogeneous reaction parameter K on concentration is outlined in Fig. 8. A reduction in the concentration is depicted for the greater counts of (K=1.1,2.1,3.1). As K increases, h(η) develops a shear layer-type structure. MWCNTs are more responsive than the SWCNTs to increasing homogeneous reaction parameter K. The effect of rate constant $K_{1}$ on h(η) is plotted in Fig. 9. Decreasing behavior in h(η) is noticed on increasing $\left(K_{1}=1.0,1.3,1.6\right)$ for scenario of single-wall and multi-wall CNTs. And solutal boundary layer thickness also decreases on boosting $\left(K_{1}=1.0,1.3,1.6\right)$. Single-wall carbon nanotubes in water-based liquid have comparatively stronger effects on solutal boundary layer thickness than the multi-walled carbon nanotubes in water-based fluid. Similar effects of single-wall carbon nanotubes over the multi-walled carbon nanotubes in terms of solutal boundary layer thickness is examined for kerosene oil-based fluid.

### 5.4 Heat transfer rate and surface drag coefficient

The impact of different emerging thermophysical parameters on Surface drag coefficient CfRex12 and rate of heat flux NuxRex12 for water-based and kerosene oil-based SWCNTs and MWCNTs are presented in Table 3, Table 4. Both friction factor coefficients as well as the rate of heat transfer decrease on increasing φ in the case of both single and multi-wall CNTs with both base fluids. Also, the declined behavior of the heat transfer rate is observed for a higher source and sink parameter in the case of both the base fluids. Whereas, increasing trends of both SWCNTs and MWCNTs in the consideration of water and kerosene oil base fluids are observed upon increasing the second-order slip parameter. Table 5 explains the validation of the presented model by comparing it with computed results in a limiting case. Great consistency is achieved, showcasing an impressive correlation.

**Table 3:** Numerical assessment of NuxRex12 for involved parameters.

NuxRex12			Water		Kerosene oil	
φ	$A^{*}$	$B^{*}$	SWCNTs	MWCNTs	SWCNTs	MWCNTs
**0.01**	2.8	0.05	−0.21081631	−0.20788143	−0.07731258	−0.07453260
**0.02**			−0.24370259	−0.23777956	−0.09668380	−0.09103650
**0.03**			−0.2772157	−0.26835749	−0.11573075	−0.10719191
	2.9		−0.21574243	−0.21276817	−0.07878498	−0.07599883
	3.0		−0.22066893	−0.21765554	−0.08025743	−0.07746472
		0.06	−0.21129177	−0.20835296	−0.07744112	−0.07612641
		0.07	−0.21176729	−0.20882472	−0.07756922	−0.07625399

**Table 4:** Numerical assessment of CfRex12 for involved parameters.

CfRex12						Water		Kerosene oil	
φ	β	Fr	$\lambda_{2}$	γ	δ	SWCNTs	MWCNTs	SWCNTs	MWCNTs
**0.01**	0.2	0.5	1.5	2.9	−2.0	−2.1820614	−2.1943876	−2.1771643	−2.3054591
**0.02**						−2.1940097	−2.2187803	−2.1808756	−2.3259926
**0.03**						−2.2067074	−2.2440611	−2.1855191	−2.3473538
	0.3					−2.1970077	−2.1963649	−2.1915798	−2.3208086
	0.4					−2.2102735	−2.1984876	−2.2043512	−2.3345242
		0.6				−2.2330207	−2.2043116	−2.2281641	−2.3561195
		0.7				−2.2838514	−2.2089269	−2.2790334	−2.4066592
			1.6			−2.2383725	−2.1953276	−2.2330217	−2.3615737
			1.7			−2.2944981	−2.1962389	−2.2887013	−2.3756789
				3.0		−2.1813122	−2.1823411	−2.2879933	−2.1919442
				3.1		−2.1805731	−2.1819872	−2.2882933	−2.1912009
					−2.1	−2.2509518	−2.1952479	−2.3621741	−2.3517025
					−2.2	−2.3197686	−2.1973451	−2.3832194	−2.3906084

**Table 5:** Comparison of tabulated results with Gireesha et al. [25] and Bashir et al. [26] for Skin friction coefficient $f^{\prime \prime }\left(0\right)$ considering F=0 by varying magnitudes of A=a/c.

A=a/c	[25]	[26]	**Present study**
0.1	−0.96938	−0.96933	−0.96921
0.2	−0.91810	−0.91811	−0.91809
0.5	−0.66723	−0.66724	−0.66231
1.0	0.90852	0.90853	0.90789
2.0	2.01750	2.01752	2.01692
3.0	4.72928	4.72930	4.72992
4.0	8.00043	8.00046	8.00082

## 6 Conclusions

The influence of inclined magnetic flux on a Darcy Forchheimer flow has been investigated in this present work. Also, velocity slip effects have been applied on the boundary of the stretchable sheet in occurrence with an h-h reaction on the SWCNTs and MWCNTs. The heating mechanism is also analyzed by considering heat source/sink effects. The following are the study's final findings:

➢ The momentum boundary layer is enhanced for the large value of the volume fraction of the nanoparticle.
➢ Fluid velocity is increased by decreasing both first and second-order velocity slip parameters.
➢ The thermal boundary layer boosts the mounting heat source and sink parameter.
➢ The induced magnetic field is decreased by upsurging the reciprocal magnetic Prandtl number.
➢ Upon increasing the homogeneous reaction parameter, the solutal boundary layer is also increased.
➢ Single-walled water-based carbon nanotube fluid has comparatively stronger effects on solutal boundary layer thickness than the multi-walled carbon nanotubes in water-based fluid and similar effects are observed in the scenario of kerosene oil-based fluid. Also, increasing trends of both SWCNTs and MWCNTs in the employment of water and kerosene oil base fluids are observed upon increasing the second-order slip parameter.
➢ Both CfRex12 and NuxRex12 drop down for increasing the volume fraction of nanoparticles.

### 6.1 Limitation of current work

The following are the limitations of the current work:

➢ This mathematical model consists of highly nonlinear PDEs, which are not possible to solve with exact methods and, therefore handled with the numerical approximations method.
➢ This current model is the Tiwari and Das model, therefore Brownian motion effects can not be computed.
➢ This present investigation is purely theoretical, without any experimental lab work. So no direct application can be quoted for the presented model

### 6.2 Future work suggestions

This current work can also be considered by adding magnetic dipole effects, introducing exponential stretched sheets, and Hall effects.

## Additional information

No additional information is available for this paper.

## Data availability statement

No data was used for the research described in the article.

## CRediT authorship contribution statement

**Seemab Bashir:** Writing – original draft. **Ibrahim M. Almanjahie:** Writing – review & editing, Validation. **Muhammad Ramzan:** Project administration, Conceptualization. **Ammara Nawaz Cheema:** Software. **Muhammad Akhtar:** Writing – review & editing. **Fatimah Alshahrani:** Writing – review & editing.

## Declaration of competing interest

The authors declare that they have no known competing financial interests or personal relationships that could have appeared to influence the work reported in this paper.

## Nomenclature

$\overset{⌢}{u},\overset{⌢}{v}$: Velocity components m/s
δ: Second-order velocity slip parameter
$C_{2}$: Concentration of heterogeneous reaction
μ: Dynamic viscosity $m^{2}/s$
$\mu_{nf}$: Nanofluid dynamic viscosity $m^{2}/s$
$D_{A}$: Coefficient of diffusion specie A
${\text{Re}}_{x}$: Local Reynolds number
Sc: Schmidt number
$D_{B}$: Coefficient of diffusion specie B
F: Darcy Forchheimer parameter
$T_{\infty }$: Ambient temperature K
$T_{w}$: Wall temperature K
$k_{n}$: Knudsen number
$\nu_{f}$: Kinematic viscosity for base fluid $m^{2}/s$
$k_{CNT}$: Thermal conductivity of CNTs $W\;m^{-1}K^{-1}$
${A_{1}}^{*}$: Constant coefficient for mean free path m
F: non-uniform inertia coefficient
β: Magnetic parameter
$\alpha_{nf}$: Nanofluid thermal conductivity $Wm^{-1}K^{-1}$
$\lambda_{2}$: Porosity parameter
$\theta_{w}$: Temperature ratio parameter
$\delta^{*}$: Rate of diffusion coefficient
$K^{*}$: Heterogeneous reaction parameter
$C_{\infty }$: Ambient concentration particles/L
Pr: Prandtl number
K: Homogeneous reaction parameter
${\overset{⌢}{H}}_{1},{\overset{⌢}{H}}_{2}$: Magnetic components
x,y: Cartesian coordinates m
T: Temperature K
$u_{w}$: Stretching velocity m/s
$a,c,H_{ο},C_{ο}$: Dimensional constant
$K_{1},K_{2}$: Rate constant
$\rho_{f}$: Density of base fluid $kg/m^{3}$
C: Concentration particles/L
φ: Volume fraction of CNTs
q*: Non-uniform heat source/sink
λ: Reciprocal magnetic Prandtl number
$k_{f}$: Thermal conductivity of base fluid $W\;m^{-1}K^{-1}$
ξ: momentum accommodation coefficient
ω: molecular mean free path
$\rho_{CNT}$: Density of CNTs $kg/m^{3}$
${B_{1}}^{*}$: Square of constant coefficient for mean free path $m^{2}$
$F_{r}$: Forchheimer parameter
$A^{*},B^{*}$: Heat source/sink space and temperature-dependent coefficients
$u_{e}$: Free stream velocity m/s
$C_{1}$: Concentration of homogeneous reaction
$q_{w}$: Wall heat flux $W/m^{2}$
A: Stretching ratio parameter m
$A_{1},B_{1}$: Chemical species
γ: First-order velocity slip parameter
$H_{e}$: Magnetic field at free stream
$\rho_{nf}$: Density of nanoparticles $kg/m^{3}$

## Appendix A

Dimensional analysis of the governing model equations is presented in this section:

(Equation (5))

$$\begin{array}{l} \overset{⌢}{u}{\overset{⌢}{u}}_{x}+\overset{⌢}{v}{\overset{⌢}{u}}_{y}-\frac{\mu_{e}}{4\pi \rho_{nf}}\left[{\overset{⌢}{H}}_{1}{\left({\overset{⌢}{H}}_{1}\right)}_{x}+{\overset{⌢}{H}}_{2}{\left({\overset{⌢}{H}}_{1}\right)}_{y}\right]=\frac{\mu_{e}}{4\pi \rho_{nf}}{\overset{⌢}{H}}_{e}{{\overset{⌢}{H}}_{1}}^{\prime }+\frac{\mu_{nf}}{\rho_{nf}}{\overset{⌢}{u}}_{yy}-\frac{\mu_{nf}}{\rho_{nf}k^{*}}\overset{⌢}{u}-F{\overset{⌢}{u}}^{2}\text{,}\\ \overset{⌢}{u}{\overset{⌢}{u}}_{x}=\frac{L}{T}\left(\frac{L}{T}\times \frac{1}{L}\right)=\frac{L}{T^{2}},\frac{\mu_{e}}{\rho_{nf}}{\overset{⌢}{H}}_{1}{\left({\overset{⌢}{H}}_{1}\right)}_{x}=\left(\frac{M}{T^{2}I}\times \frac{M}{LT^{2}I}\times \frac{T^{2}I^{2}}{ML}\times \frac{L^{3}}{M}\right)=\frac{L}{T^{2}}\text{,}\\ \frac{\mu_{e}}{\rho_{nf}}{\overset{⌢}{H}}_{2}{\left({\overset{⌢}{H}}_{1}\right)}_{y}=\left(\frac{M}{T^{2}I}\times \frac{M}{LT^{2}I}\times \frac{T^{2}I^{2}}{ML}\times \frac{L^{3}}{M}\right)=\frac{L}{T^{2}},F{\overset{⌢}{u}}^{2}=\left(\frac{1}{L}\times \frac{L^{2}}{T^{2}}\right)=\frac{L}{T^{2}}\text{,}\\ \frac{\mu_{nf}}{\rho_{nf}}{\overset{⌢}{u}}_{yy}=\left(\frac{L^{2}}{T}\times \frac{L}{T}\times \frac{1}{L^{2}}\right)=\frac{L}{T^{2}},\frac{\mu_{e}}{\rho_{nf}}{\overset{⌢}{H}}_{e}{{\overset{⌢}{H}}_{1}}^{\prime }=\left(\frac{M}{T^{2}I}\times \frac{M}{LT^{2}I}\times \frac{T^{2}I^{2}}{ML}\times \frac{L^{3}}{M}\right)=\frac{L}{T^{2}}\text{,}\\ \overset{⌢}{v}{\overset{⌢}{u}}_{y}=\frac{L}{T}\left(\frac{L}{T}\times \frac{1}{L}\right)=\frac{L}{T^{2}},\frac{\mu_{nf}}{\rho_{nf}k^{*}}\overset{⌢}{u}=\left(\frac{L^{2}}{T}\times \frac{L}{T}\times \frac{1}{L^{2}}\right)=\frac{L}{T^{2}}\text{,} \end{array}$$

(Equation (6))

$$\begin{array}{l} \overset{⌢}{u}{\left({\overset{⌢}{H}}_{1}\right)}_{x}-{\overset{⌢}{H}}_{1}{\overset{⌢}{u}}_{x}+\overset{⌢}{v}{\left({\overset{⌢}{H}}_{2}\right)}_{y}-{\overset{⌢}{H}}_{2}{\overset{⌢}{u}}_{y}=\mu_{e}{\left({\overset{⌢}{H}}_{1}\right)}_{yy}\text{,}\\ \overset{⌢}{u}{\left({\overset{⌢}{H}}_{1}\right)}_{x}=\frac{L}{T}\left(\frac{M}{T^{2}I}\times \frac{1}{L}\right)=\frac{M}{T^{3}I},\overset{⌢}{v}{\left({\overset{⌢}{H}}_{2}\right)}_{y}=\frac{L}{T}\left(\frac{M}{T^{2}I}\times \frac{1}{L}\right)=\frac{M}{T^{3}I},{\overset{⌢}{H}}_{1}{\overset{⌢}{u}}_{x}=\left(\frac{M}{T^{2}I}\times \frac{L}{T}\times \frac{1}{L}\right)=\frac{M}{T^{3}I}\text{,}\\ {\overset{⌢}{H}}_{2}{\overset{⌢}{u}}_{y}=\left(\frac{M}{T^{2}I}\times \frac{L}{T}\times \frac{1}{L}\right)=\frac{M}{T^{3}I},\mu_{e}{\left({\overset{⌢}{H}}_{1}\right)}_{yy}=\left(\frac{L^{2}}{T}\times \frac{M}{T^{2}I}\times \frac{1}{L^{2}}\right)=\frac{M}{T^{3}I}\text{,} \end{array}$$

(Equation (7))

$$\begin{array}{l} \overset{⌢}{u}{\overset{⌢}{T}}_{x}+\overset{⌢}{v}{\overset{⌢}{T}}_{y}=\alpha_{nf}{\overset{⌢}{T}}_{yy}+\frac{q*}{{\left(\rho c_{p}\right)}_{nf}}\text{,}\\ \overset{⌢}{u}{\overset{⌢}{T}}_{x}=\frac{L}{T}\left(\frac{\theta }{L}\right)=\frac{\theta }{T},\overset{⌢}{v}{\overset{⌢}{T}}_{y}=\frac{L}{T}\left(\frac{\theta }{L}\right)=\frac{\theta }{T},\alpha_{nf}{\overset{⌢}{T}}_{yy}=\frac{L^{2}}{T}\left(\frac{\theta }{L^{2}}\right)=\frac{\theta }{T}\text{,}\\ \frac{q*}{{\left(\rho c_{p}\right)}_{nf}}=\left(\frac{ML^{2}}{T^{3}L^{3}}\times \frac{\theta T^{2}L^{3}}{ML^{2}}\right)=\frac{\theta }{T}\text{,} \end{array}$$

(Equation (8))

$$\begin{array}{l} \overset{⌢}{u}{\left(C_{1}\right)}_{x}+\overset{⌢}{v}{\left(C_{1}\right)}_{y}=D_{A}{\left(C_{1}\right)}_{yy}-K_{1}C_{1}{C_{2}}^{2}\text{,}\\ \overset{⌢}{u}{\left(C_{1}\right)}_{x}=\frac{L}{T}\left(\frac{Mol}{L^{3}}\times \frac{1}{L}\right)=\frac{Mol}{L^{3}T},\overset{⌢}{v}{\left(C_{1}\right)}_{y}=\frac{L}{T}\left(\frac{Mol}{L^{3}}\times \frac{1}{L}\right)=\frac{Mol}{L^{3}T},D_{A}{\left(C_{1}\right)}_{yy}=\frac{L^{2}}{T}\left(\frac{Mol}{L^{3}}\times \frac{1}{L^{2}}\right)=\frac{Mol}{L^{3}T}\text{,}\\ K_{1}C_{1}{C_{2}}^{2}={\left(\frac{Mol}{L^{3}}\right)}^{-2}T^{-1}\times \frac{Mol}{L^{3}}\times {\left(\frac{Mol}{L^{3}}\right)}^{2}=\frac{Mol}{L^{3}T}\text{,} \end{array}$$

Similarly, dimension for Equation (9) is calculated.
